# Huqi San-Evoked Rat Colonic Anion Secretion through Increasing CFTR Expression

**DOI:** 10.1155/2015/301640

**Published:** 2015-07-28

**Authors:** Xiaowei Xue, Zhengming Shi, Wen Wang, Xiaotong Yu, Ping Feng, Min Zhang, Xuejiang Wang, Jingdong Xu

**Affiliations:** ^1^Department of Physiology and Pathophysiology, School of Basic Medical Science, Capital Medical University, Beijing 100069, China; ^2^Department of Pathology, Peking Union Medical College Hospital, Chinese Academy of Medical Sciences & Peking Union Medical College, Beijing 100730, China; ^3^Department of General Surgery, Beijing Jishuitan Hospital, Beijing 100035, China; ^4^Department of Pharmacology, Xuanwu Hospital of Capital Medical University, Beijing 100053, China; ^5^Alzheimer's Disease Center, Beijing Institute for Brain Disorders, Capital Medical University, Beijing 100069, China; ^6^Department of Statistics, Purdue University, West Lafayette, IN, USA

## Abstract

Huqi San (HQS) is a Chinese herbal preparation of eight medicinal herbs that promote diuresis, detoxification, blood circulation, and cholestasis. Defects in transporter expression and function can cause cholestasis and jaundice. However, the mechanism of the cholestasis underlying HQS effects, especially on the gastrointestinal tract ion secretion, has not been elucidated. Real-time RT-PCR and Western blotting were used to study the expression and localization of cystic fibrosis transmembrane conductance regulator (CFTR) and *α*-ENaC in rat alimentary tract, and then the effect of HQS on the ion transport in rat distal colon mucosa was investigated using the short-circuit current (*I*_SC_) technique. The results showed that pretreatment with HQS significantly enhanced mRNA transcripts and protein content of CFTR in liver and distal colon but not *α*-ENaC in alimentary organs. HQS increases *I*_SC_ and decreases the transepithelial resistance. Pretreatment with epithelial Na^+^ channel blocker did not affect the *I*_SC_ responses elicited by HQS, but removal of extracellular Cl^−^ or pretreatment with Cl^−^ channel or Na^+^-K^+^-2Cl^−^ cotransporter blocker inhibited HQS-elicited *I*_SC_ responses. These findings demonstrated that HQS, RA, and RP can stimulate Cl^−^ secretion in the distal colon by increasing the mRNA transcripts and protein content of CFTR in liver and distal colon.

## 1. Background

Huqi San (HQS) is a Chinese herbal preparation of eight medicinal herbs, including Ramulus Visci, Radix Astragali seu Hedysari, Radix Curcumae, Radix Salviae Miltiorrhizae, Spica Prunellae, Semen Persicae, Semen Cuscutae, and Radix Sophorae Flavescentis, purchased from Tongrentang (Beijing, China) and authenticated by Professor Wen Wang, a botanist at Xuanwu Hospital, Beijing, China. HQS supplements qi and tonifies the kidney. HQS, especially its principal drug Ramulus Visci alkali (RA), the major active constituent of mistletoe extracts, is widely used for the treatment of tumour [1], hypertensive rats, and renal hypertensive dogs [2]. Previous studies showed that RA can inhibit Ca^2+^ mobilization from intracellular stores [3], block and reverse hepatocarcinogenesis [1], gynecological and breast cancer treatment [4], and even enhance immunosurveillance to prevent intestinal infections or other intestinal pathologies by the induction of cytokines in intestinal epithelial cells [5]. As an adjuvant, polysaccharides of Ramulus Visci (RP) can inhibit cancer cell proliferation and promote cancer cell apoptosis* in vivo* [6]. Our previous experiment has confirmed the effect of HQS on hepatocarcinogenesis [7]. Laboratory tests revealed hepatic cell damage with cholestasis, lipid abnormalities, and hypocholesterolemia with cystic fibrosis associated with liver disease as the only manifestation of cystic fibrosis [8]. Cholangiocytes alkalinize and dilute canalicular bile through the secretion of a bicarbonate rich fluid. Cystic fibrosis transmembrane conductance regulator (CFTR), a cAMP-regulated chloride channel expressed in biliary tract, is the major driving force for this ductular secretion [9, 10]. Both human and animal studies have provided evidence that any impairment in the expression and/or function of these different hepatobiliary transporters may lead to cholestatic disorders [11]. The disruption and dysregulation of this excretory pathway may result in cholestasis [12] and lead to intrahepatic accumulation of bile acids and other toxic compounds with progression of hepatic pathological changes [13]. Although the transcriptional regulation of hepatic organic anion transporters by liver-enriched hepatocyte nuclear factors and ligand activated nuclear receptors is the key to understand the molecular mechanisms of cholestasis [14], the transporter changes at a transcriptional level may represent potential targets for therapy [14]. In this study we investigate the effects of HQS, RA, and RP on hepatic organic anion transporter regulation in the liver, distal colon, and pancreas of rat.

## 2. Materials and Methods

### 2.1. Preparation of HQS

*HQS* was prepared from eight medicinal herbs by soaking the herbs, which include Hujisheng 1800 g (*Viscum coloratum *(Komar.) Nakai, Shanxi), Huangqi 1600 g (*Astragalus membranaceus *(Fisch.)* Bge. var. mongholicus *(Bge.)* Hsiao*, Gansu), Yujin 1200 g (*Curcuma wenyujin *Y. H. Chen and C. Ling., Sichuan), Danshen 800 g (*Salvia miltiorrhiza *Bge., Gansu), Xiakucao 800 g (*Prunella vulgaris *L., Henan), Taoren 600 g (*Prunus persica* (L.) Batsch, Beijing), Buguzhi 1200 g (*Psoralea corylifolia *L., Yunnan), and Kushen 800 g (*Sophora flavescens *Ait., Jiangsu) which were extracted with 95% ethanol in the proportion of 1 : 8 (w/v) for 2 h and repeated twice, and then the ethanol extracts were concentrated with a rotary evaporator (RE-52AA, Shanghai, China) and lyophilized (Thermo Savant, USA). The extracts were dissolved in water and extracted with petroleum ether. Subsequently, the defatted fraction was extracted with ethyl acetate and then concentrated to ointment under reduced pressure. The ointment was subjected to vacuum drying to form extractum which was then crushed into pieces, granulated, and stored in 4°C refrigerator until use. The HQS granules were diluted with water in the concentration 0.38 g dry grains/mL [15].

### 2.2. Preparation of Alkalies and Polysaccharides

The regular experiment method was performed for qualitative analyses of the alkalies or polysaccharides in Ramulus Visci. Ramulus Visci was ground to powder. Before soaking in acidity aqueous for 48 h, alkalies in Ramulus Visci (RA) were proceeded to precipitate with the alkali and then the insoluble were filtered. The partial precipitates were achieved using different alkalinity ratios to get the total alkali, and the polysaccharides of Ramulus Visci (RP) were prepared according to the previous report [16].

### 2.3. Solutions and Reagents

Hank's balanced salt solution (HBSS), glibenclamide, amiloride hydrochloride, bumetanide, and forskolin are from Sigma (St. Louis, MO, USA). The stock solutions of glibenclamide, forskolin, and bumetanide were prepared in dimethyl sulfoxide (DMSO). Final concentrations of DMSO never exceeded 0.1% (v/v). Preliminary experiments indicated that the vehicle did not change any baseline electrophysiological parameters.

Krebs-Henseit solution (K-HS) includes the following ingredients (mM): NaCl, 117; KCl, 4.5; CaCl_2_, 2.5; MgCl_2_, 1.2; NaHCO_3_, 24.8; KH_2_PO_4_, 1.2; glucose, 11.1. In Cl^−^-free solution, NaCl, KCl, and CaCl_2_ were replaced by sodium gluconate, potassium gluconate, and calcium gluconate, respectively. The solution was gassed with 95% O_2_-5% CO_2_ (V/V), pH 7.4, at temperature 37°C.

### 2.4. Tissue Preparation

Animal protocols followed guidelines established by the NIH and were approved by Animal Care and Use Committee, Capital Medical University. Male Sprague-Dawley rats (Laboratory Animal Services Center, Capital Medical University) ranging from 135 to 150 g (6 weeks old) had free access to standard rodent laboratory food and water until the day of the experiments. Eighty rats were equally divided (20 rats/group) into HQS therapeutic group (8 g/kg body weight), RA therapeutic group (10 mg/kg body weight), RP therapeutic group (10 mg/kg body weight), and control group with the physiological saline. All the drugs were given through intragastric administration for seven days* in vivo* experiment. The animals were killed by cervical dislocation. The distal colon was removed and defined as the* ca.* 7 cm long segment proximal to the lymph node (typically situated 3 cm apart from the anus). Then the distal colon was divided into 4 segments, which were cut along the mesenteric border into a flat sheet and flushed with ice-cold Kreb's-Henseit solution (K-HS). The tissue was pinned flat with the mucosal side down in a Sylgard-lined petri dish containing ice-cold oxygenated solution. The colon was longitudinally cut close to the mesentery, and the serosal muscle layers were carefully stripped away by blunt dissection to obtain a mucosa preparation. All the herbs and the routine drugs were supplied by means of directly apical or basolateral side in Ussing chamber system.

### 2.5. Short-Circuit Current Measurement

The short-circuit current was measured* in vitro* in Ussing chambers. Flat sheet of colonic mucosa preparations was mounted between two halves of modified Ussing chambers, in which the total cross-sectional area was 0.5 cm^2^. The mucosal and serosal surfaces of tissue were bathed with 5 mL K-HS by recirculation from a reservoir maintained at 37°C during the experiments. The K-HS was bubbled with 95% O_2_-5% CO_2_ to maintain the pH of the solution at 7.4. Drugs could be added directly to the apical or basolateral side of mucosa. Responses were continuously recorded by computer. Transepithelial potential difference for every colonic mucosa was measured by the Ag/AgCl reference electrodes (Physiologic Instruments, P2020S) connected to a preamplifier that was in turn connected to a voltage-clamp amplifier VCC MC6 (Physiologic Instruments). The change in *I*_SC_ was calculated using the value before and after simulation and was normalized as current per unit area of epithelial tissue (*μ*A·cm^−2^), which allowed the curve area for 15 minutes to be calculated (*μ*A·min). The change in current in response to the applied potential was used to calculate the transepithelial resistance of the monolayer by Ohm's law. Experiments were repeated in different batches of colon mucosa to ensure that the data were reproducible. Positive *I*_SC_ corresponded to the movement of anions from the serosal to mucosal compartments or movement of cations from the mucosal to serosal compartments or a combination of both.

### 2.6. Preparation of RNA and cDNA

The distal colonic, liver, and pancreas tissues were collected in phosphate buffered saline (PBS; 0.9% NaCl in 0.01 M sodium phosphate buffer, pH 7.4), which had been treated with 0.1% diethylpyrocarbonate (DEPC-PBS). After the wall of each piece of tissue had been opened, the tissue was cleaned with DEPC-PBS and transferred to Trizol (Invitrogen) for extraction of total RNA, which was isolated according to the manufacturer's instructions and stored at −80°C for later use. Samples of cDNA were generated by reverse transcription with 5 *μ*g total RNA, 50 ng random hexamer primers, and 10 nM dNTPs, incubated at 65°C for 5 min, and placed on ice for at least 1 min, with the addition of 40 URNase OUT, 200U SuperScript III RT, 10 mM dithiothreitol, and 5 mM MgCl_2_ (Invitrogen), in a 20 *μ*L reaction volume. Following brief centrifugation, the reactions were incubated at 50°C for 50 min and then at 70°C for 15 min. The completed reverse transcription reactions were stored at −20°C and used for the polymerase chain reaction (PCR) without further treatment.

### 2.7. Real-Time RT-PCR and Sequencing

Total RNA of the liver, colon, and pancreas was extracted using the Trizol reagent (Invitrogen, Carlsbad, CA, USA) according to the protocol of the manufacturer. Primers for CFTR were designed according to the rat mRNA sequence of CFTR (Sangon, Shanghai, China): 5′-cgc agg ttc tca gtg gac gat gcc-3′ (forward) and 5′-cct caa cca gaa aaa cca gca cgc-3′ (reverse) (estimated amplicon size: product length 269 bp). mRNA sequence of *α*-ENaC (Sangon, Shanghai, China): 5′-cag-ggt-gat-ggt-gca-tgg-3′ (forward) and 5′-cca cgc cag gtc aag-3′ (reverse) (estimated amplicon size: product length 239 bp), GAPDH forward primer: 5′-tgg agt cta ctg gcg tct-3′, reverse primer: 5′-agt gag ctt ccc gtt cag-3′ was used as an internal control (Table 1). All RT-qPCRs were performed with a Bio-Rad CFX 96 Real-time Detection Systems (Bio-Rad, Hercules, CA, USA). Protocols for all cycle RT-qPCRs were adjusted to the same thermal profile. Data were collected during the annealing phase.

### 2.8. Western Blotting

Tissue was harvested from the rat liver, distal colon, and pancreas, washed with PBS, and homogenized in 300 *μ*L cold lysis buffer, pH 7.5, containing Nonidet P-40 (1%), TRIS-HCl (10 mM, pH 8.0), EDTA (1.0 mM), NaCl (150 mM), EGTA (2.0 mM), 10% SDS (0.1%), sodium orthovanadate (1 mM), deoxycholic acid (0.5%), phenylmethanesulfonyl fluoride (1.0 mM), aprotinin (5 *μ*g/mL), and leupeptin (5 *μ*g/mL), all purchased from Sigma. Total tissue homogenates were sonicated to dissolve completely and then centrifuged at 12,000 rpm for 30 min at 4°C to separate the membrane-containing fraction (pellet) from the cytosol. Proteins (100 *μ*g) were separated by 10% SDS-polyacrylamide gel electrophoresis. The separated proteins were electroblotted onto nitrocellulose membrane (NC membrane, Millipore), and then the membrane was washed for 10 min with TBST (20 mM TRIS-HCl, pH 7.5, containing 0.15 M NaCl and 0.05% Tween 20) and immersed in blocking buffer containing 5% nonfat dry milk in TBST for 1 h at room temperature. The blot was washed with TBST and finally incubated overnight at 4°C with polyclonal primary antibodies to CFTR and *α*-ENaC (Affinity Bio Reagents, USA; diluted 1 : 500 in 5% nonfat dry milk). After being washed in TBST, the blot was incubated with secondary antibody to rabbit lgG (Zhongshan Goldenbridge, China) for 1 h at room temperature. The blot was finally washed with TBST, scanned by infrared rays with the Odyssey Infrared Imager (LI-COR, Nebraska, USA), and analyzed by Odyssey software (version 1.2).

### 2.9. Statistical Analysis

All values were expressed as means and standard error of mean (S.E.M.), and *n* was the number of animals in each experiment. All data were analyzed using the GraphPad Prism software 5.0 package (GraphPad Software Inc., San Diego, CA, USA). The increase in *I*_SC_ was quantified by subtracting the peak of an *I*_SC_ response from its respective baseline value before drug administration. The differences between control and treatment means were analyzed using Student's paired or unpaired *t*-test when appropriate. The differences among groups were analyzed using a one-way analysis of variance followed by Dunnett's multiple comparison. A *P* value of less than 0.05 was considered statistically significant.

## 3. Results 

### 3.1. Effects of HQS, RP, and RA on the mRNA Expressions of CFTR and *α*-ENaC

**Figure 1:**
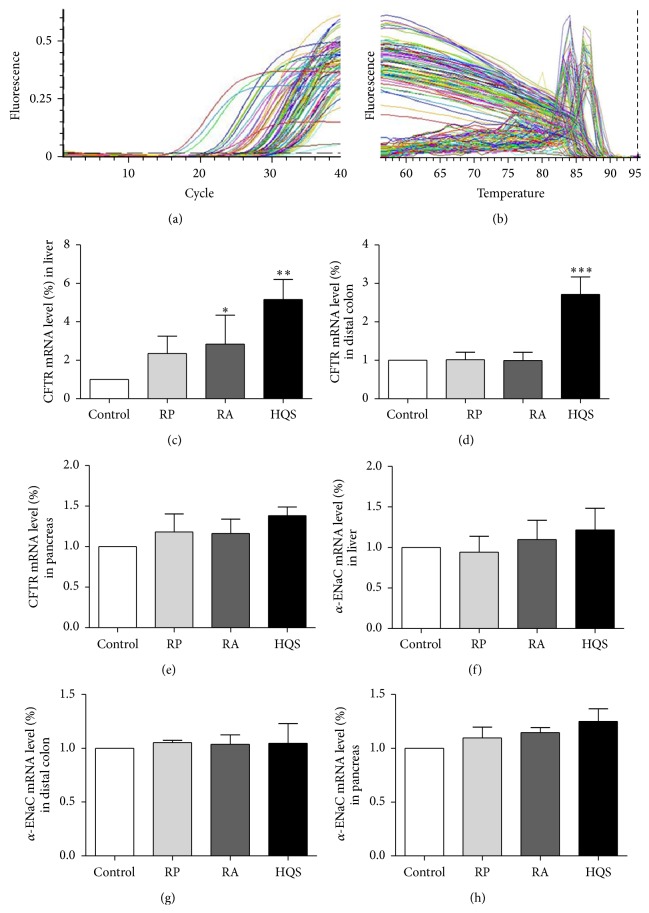
Real-time RT-PCR analysis of mRNA expression of CFTR and *α*-ENaC in rat liver, distal colon, and pancreas. (a) Continuous monitoring of fluorescence emission during cycle PCR of template DNA from strains tested. The number of samples for liver, pancreas, and distal colon is 35 and 40, respectively. The error bars represent standard error. (b) Representative different tissues experiment by melting analysis based on real-time PCR using SYBR Green I. The melting point peaks were clearly separated among liver, pancreas, and distal colon using SYBR Green I at 2.5 mM MgCl_2_. Real time RT-PCR results with products of CFTR found in rat liver, pancreas, and distal colon quantitative analysis of CFTR expression pretreatment with RP, RA, and HQS, respectively, and GAPDH (internal marker) ratio shown. RA and HQS could enhance the CFTR expression in rat liver and distal colon but not in pancreas ((c)–(e)). Quantitative analysis of *α*-ENaC expression pretreatment with RP, RA, and HQS, respectively, and GAPDH (internal marker) ratio were shown. There is no statistically significant change in liver, pancreas, or distal colon ((f)–(h)). Data analysis was used with one-way analysis of variance followed by Dunnett's multiple comparison. Values are represented as means ± SEM; ^*∗*^ *P* < 0.05, ^*∗∗*^ *P* < 0.01.

In order to investigate whether the effects of HQS, RP, or RA on the depressed liver energy and cholestasis were related to the expressions of CFTR and *α*-ENaC, we used real-time RT-PCR analysis to examine the expression levels of CFTR and *α*-ENaC in liver, pancreas, and colon (Figures 1(c)–1(e)). The quantitative analyses showed that the expression levels of CFTR but not *α*-ENaC were significantly elevated after HQS, RP, or RA pretreatment, respectively. The CFTR mRNA level (%) in liver increased to 2.83 ± 0.62 in RA (*n* = 6, *P* < 0.05) and to 5.15 ± 0.42 in HQS (*n* = 6, *P* < 0.001) but not in RP (Figure 1(c)). The CFTR mRNA level (%) in colon was increased to 2.72 ± 0.45 in HQS (*n* = 6, *P* < 0.001) (Figure 1(d)) but no obvious changes in RA or RP. However, the CFTR in pancreas has no obvious changes after treatment with HQS, RA, or RP (Figure 1(e)). As shown in Figures 1(f)–1(h), *α*-ENaC has no significant change in liver, pancreas, or colon after treatment with HQS, RA, or RP.

### 3.2. Effects of HQS on the Protein Expression of CFTR and *α*-ENaC

**Figure 2:**
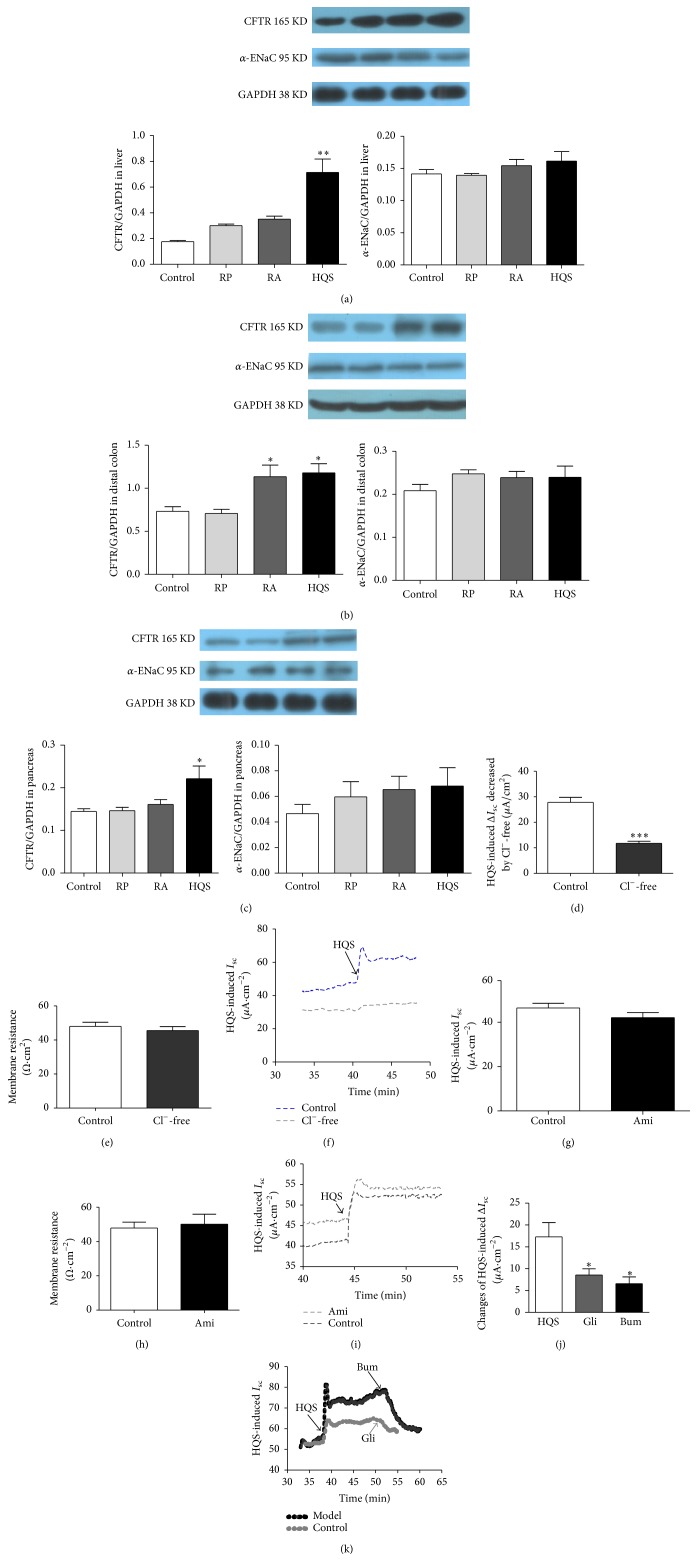
Western blot confirmed the expression of CFTR and *α*-ENaC in rat liver, distal colon, and pancreas. Densitometric analysis of CFTR and *α*-ENaC protein levels (normalized to GAPDH) in rat liver. HQS (1.0 mg/mL) can significantly increase the expression of CFTR in liver (a). Densitometric analysis of CFTR and *α*-ENaC protein levels (normalized to GAPDH) in rat distal colon. HQS (1.0 mg/mL) and RA can significantly increase the expression of CFTR in colon (b). Densitometric analysis of CFTR and *α*-ENaC protein levels (normalized to GAPDH) in rat pancreas. HQS (1.0 mg/mL) can significantly increase the expression of CFTR in pancreas (c). *α*-ENaC has no conspicuous changes across different groups ((a)–(c)). (d) Effects of Cl^−^ replacement and transporter inhibitors on the HQS *I*_SC_. The bar graph illustrates the effects of removing extracellular Cl^−^ on the HQS *I*_SC_ and transepithelial resistance (e). Representative traces of HQS-evoked Cl^−^ flux in the absence and presence of Cl^−^. Arrows indicate the time of the response to HQS (1.0 mg/mL) added basal in normal and Cl^−^-free K-H solutions (f). The bar graph illustrates the effects of apical addition of amiloride (10 *μ*mol/L) on the *I*_SC_. HQS (1.0 mg/mL) added basal in normal and Na^+^ channel blocker, amiloride (10 *μ*mol/L) (g). The bar graph illustrates of the amiloride on the transepithelial resistance of HQS (1.0 mg/mL) (h). Representative traces of HQS-evoked *I*_SC_ in the absence and presence of amiloride (100 *μ*M). Arrowheads indicate the time of HQS addition (i). The bar graph illustrates the application of glibenclamide (1 mmol/L, apical), an inhibitor of the CFTR transporter or bumetanide (100 *μ*mol/L, basal), and the Na^+^-K^+^-2Cl^−^ cotransporter on HQS-induced *I*_SC_ comparison of HQS- (1.0 mg/mL) induced *I*_SC_ (j). Representative traces of *I*_SC_ recording with arrows indicating the time for the apical application of glibenclamide (1 mm/L) or basolateral application of bumetanide (100 *μ*mol/L), respectively, and addition of HQS (1.0 mg/mL) (k). Data analysis was used with one-way analysis of variance followed by Dunnett's multiple comparison. Values are represented as mean ± SEM; ^*∗*^ *P* < 0.05, ^*∗∗*^ *P* < 0.01.

Western blotting was performed to investigate CFTR and *α*-ENaC protein content in gastrointestinal tract. We probed lysates from liver, distal colon, and pancreas with anti-CFTR and *α*-ENaC antibody (Figures 2(a)–2(c)). The immunoblots were located at the same level as their corresponding positive controls. The immunoblot detected with CFTR and *α*-ENaC antibody was 165 KD and 95 KD which was in the range reported for CFTR and *α*-ENaC protein previously. As expected, the content levels of CFTR protein significantly increased in the liver from 0.18 ± 0.02 to 0.64 ± 0.22 (*n* = 6, *P* < 0.05, about 255.5%), distal colon from 0.66 ± 0.11 to 1.05 ± 0.26 (*n* = 6, *P* < 0.05, about 59.1%), and pancreas from 0.14 ± 0.02 to 0.22 ± 0.08 (*n* = 6, *P* < 0.05, about 57.1%) in the HQS group more than that in the control. However, there was no significant difference in the other groups, except RA in colon group. Meanwhile, *α*-ENaC protein contents have no significant changes in all tissues (shown in Table 2 and Figures 2(a)–2(c)).

In order to reconfirm the ion species involved in the HQS-induced *I*_SC_, Cl^−^ was removed from the bathing solution. The HQS response significantly decreased in Cl^−^-free solution (Figures 2(d) and 2(e)), from 27.81 ± 1.98 *μ*A/cm^2^ (*n* = 12) to 11.73 ± 0.88 *μ*A/cm^2^ (*n* = 12, *P* < 0.001, 57.82%), indicating Cl^−^-dependence of the HQS-induced current, and the transmembrane resistance has no obvious changes, while pretreatment with 10 mM amiloride (Ami), an epithelial Na^+^ channel blocker, had no effect on the HQS-induced Δ*I*_SC_ (*n* = 13, *P* > 0.05; Figures 2(g)–2(i)), which excluded the involvement of Na^+^ absorption. The effect of the Cl^−^ channel blocker, glibenclamide, on the HQS-induced *I*_SC_ was also examined. As shown in Figures 2(j) and 2(k), apically applied glibenclamide (1 mM) reduced the HQS-induced Δ*I*_SC_ response by 29.9% from 62.67 ± 4.09 *μ*A/cm^2^ to 48.25 ± 1.38 *μ*A/cm^2^ (*n* = 6, *P* < 0.01). Basolateral addition of 100 mM bumetanide, which is a strong inhibitor of the Na^+^-K^+^-2Cl^−^ cotransporter, reduced the HQS-induced *I*_SC_ more than 37.6% (*n* = 6, *P* < 0.01) as shown in Figures 2(j) and 2(k).

### 3.3. HQS-Induced **I**_SC_ Responses

**Figure 3:**
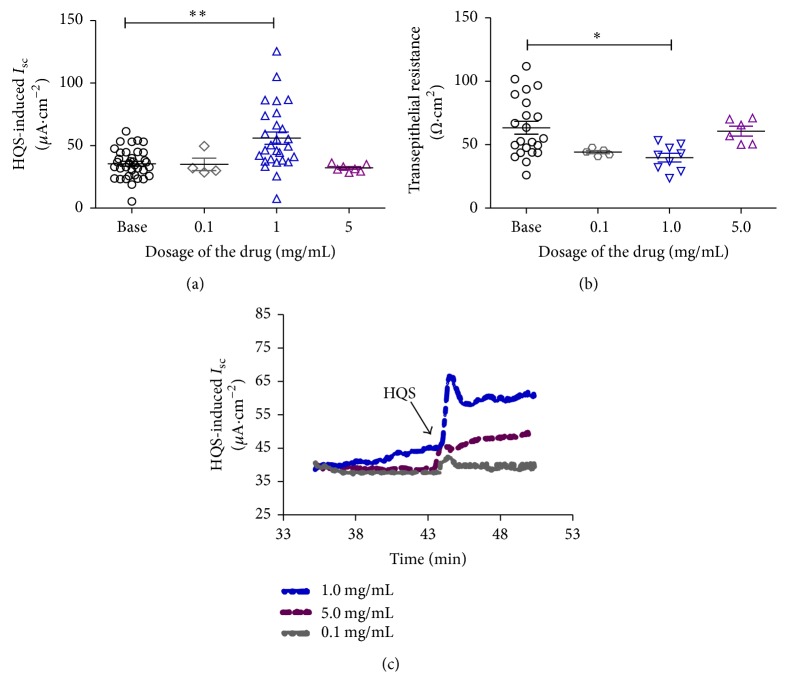
Concentration-response curve for HQS-induced response in the mucosa/submucosa preparations of the rat distal colon. Application of different dosage of HQS (0.1, 1.0, and 5.0 mg/mL) on the *I*_SC_ responses (a). The scattergram illustrates the effect of basolateral application of different concentrations of HQS on the transepithelial resistance. At the dosage of HQS (1.0 mg/mL), the colonic transepithelial resistance was significantly reduced by 58.33% (*P* < 0.05, *n* = 6) (b). Representative traces of basolateral addition of HQS-evoke *I*_SC_ responses at the concentration of 0.1, 1.0, and 5.0 mg/mL. Arrowheads indicate the time of HQS addition (c). Unpaired *t*-test was used. Values are represented as means ± SEM; ^*∗*^ *P* < 0.05, ^*∗∗*^ *P* < 0.01.

Given that HQS can enhance the expression of CFTR protein in the liver, pancreas, and colon, a mucosal preparation of the rat distal colon was chosen as a model to study the colonic secretion and analyze the potential mechanism by improving the bile from the gallbladder [17]. After the freshly isolated rat colonic mucosa had been equilibrated for 30 min, the basolateral addition of HQS produced a transient increase in *I*_SC_ that lasted approximately 10 min. The effect of HQS was concentration independent. As shown in Figure 3(a), the *I*_SC_ increase evoked by HQS at a dosage of 0.1, 1.0, and 5.0 mg/mL was from 35.49 ± 1.95 *μ*A/cm^2^to 35.03 ± 4.90 *μ*A/cm^2^ (*n* = 9, *P* > 0.05), 56.03 ± 4.95 *μ*A/cm^2^(*n* = 27, *P* < 0.01), and 32.14 ± 1.10 *μ*A/cm^2^(*n* = 8, *P* > 0.05) about 8.48%, 57.88%, or 1.69%, respectively. The HQS-induced *I*_SC_ response was accompanied by a significant dose-independent decrease in transepithelial resistance from 63.33 ± 5.06 Ω·cm^2^ to 54.17 ± 1.20 Ω·cm^2^ (*n* = 12, *P* > 0.05), 39.79 ± 3.38 Ω·cm^2^ (*n* = 9, *P* < 0.01), and 60.72 ± 3.88 Ω·cm^−2^ (*n* = 6, *P* > 0.05) at the dosage of 0.1, 1, and 5 mg/mL by 15.90%, 37.20%, and 4.12%, respectively, which indicated that mucosa conductance might be activated at the dosage of 1 mg/mL. However, at a dosage of 5 mg/mL, HQS did not affect significantly the *I*_SC_ and transepithelial resistance as compared with the baseline (Figures 3(a)–3(c)).

### 3.4. RA and RP-Induced **I**_SC_ Responses

**Figure 4:**
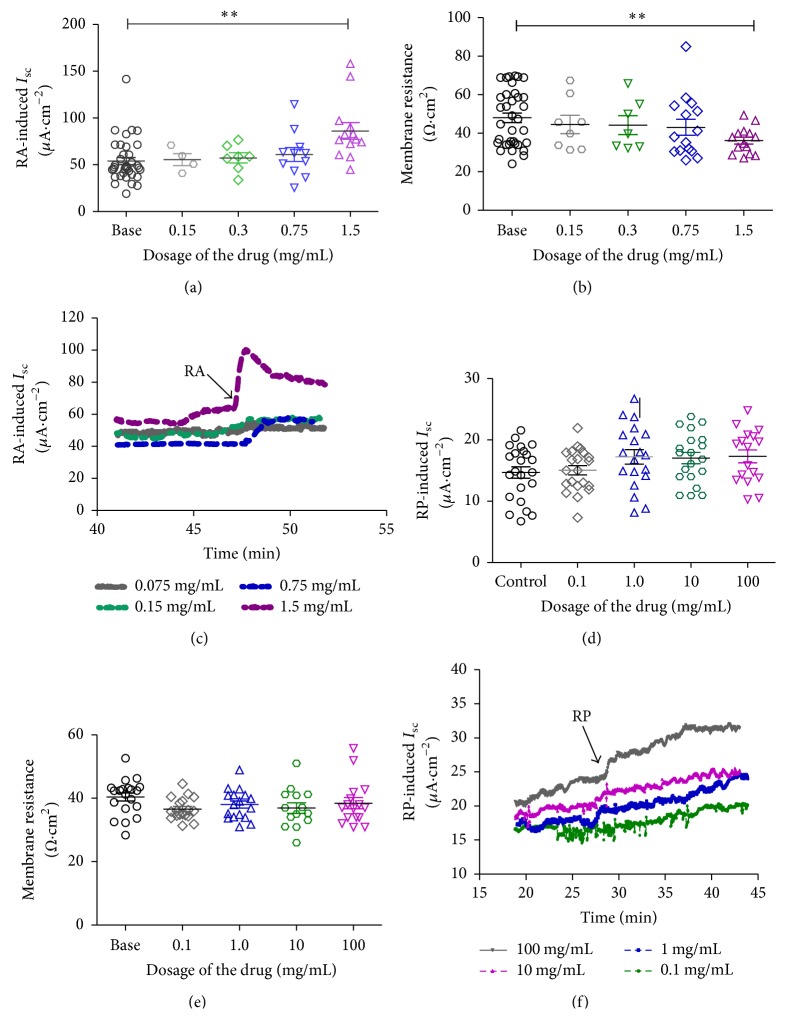
Effects of the RA and RP on the *I*_SC_ in the mucosa/submucosa preparations of the rat distal colon. The scattergram illustrates the application of different concentrations of RA (0.15, 0.3, 0.75, and 1.5 mg/mL) on the *I*_SC_ responses (a). Summary of the effects of different concentration of RA on the transepithelial resistance in the dosage of RA (1.5 mg/mL) significantly reduced colonic transepithelial resistance (b). The effect of different concentrations of RA (0.15, 0.3, 0.75, and 1.5 mg/mL) on the distal colon mucosa *I*_SC_. Arrowheads indicate the time of RA addition (c). The scattergram illustrates the application of different concentrations of RP (0.1, 1.0, 10.0, and 100 mg/mL) on the *I*_SC_ responses (d). Summary of the effects of different concentration of RP on the transepithelial resistance higher dosage of RP (1.0, 10.0, and 100.0 mg/mL) had no significant changes on colonic transepithelial resistance (e). The effect of different concentrations of RP (0.1, 1.0, 10.0, and 100.0 mg/mL) on the distal colon mucosa *I*_SC_. Arrowheads indicate the time of RP addition (f). Unpaired *t*-test was used. Data represents mean ± S.E.M., ^*∗*^ *P* < 0.05,  ^*∗∗*^ *P* < 0.01 when compared with control group alone.

As shown in Figure 2(b), RA could enhance the expression of CFTR protein in colon from 0.66 ± 0.11 to 1.05 ± 0.26 (*n* = 6, *P* < 0.05, about 59.1%) but not in liver and pancreas. So RA-induced *I*_SC_ response was observed. The result showed that RA in 0.75 mg/mL or 1.50 mg/mL increased *I*_SC_ response about 13.10% or 59.89% from 53.85 ± 3.82 *μ*A/cm^2^ to 60.90 ± 7.39 *μ*A/cm^2^ (*n* = 11, *P* > 0.05) and 86.10 ± 8.91 *μ*A/cm^2^ (*n* = 13, *P* < 0.01) and with the decrease in transepithelial resistance about 24.6% from 48.01 ± 2.48 Ω·cm^2^ to 36.2 ± 1.84 Ω·cm^2^ (*n* = 14, *P* < 0.01) in the dosage of 1.50 mg/mL but not in other dosage groups (Figure 4(c)).

As shown in Figure 4(d), RP could not induce *I*_SC_ increasing in the dosage of 1.0, 10, and 100 mg/mL about 17.36%, 16.00%, and 17.90%, respectively, from 14.69 ± 0.92 *μ*A/cm^2^ (*n* = 15) to 17.04 ± 0.92 *μ*A/cm^2^ (*n* = 15, *P* > 0.05) and 17.32 ± 1.05 *μ*A/cm^2^ (*n* = 16, *P* > 0.05). It also showed that RP might have no effect on transepithelial resistance from 40.39 ± 1.26 Ω·cm^2^ to 38.00 ± 1.44 Ω·cm^2^(*n* = 18, *P* > 0.05), 36.91 ± 1.67 Ω·cm^2^ (*n* = 15, *P* > 0.05), and 38.42 ± 1.75 Ω·cm^2^ (*n* = 17, *P* > 0.05), respectively. All these results indicated that ion channels might be partly activated and the mucosal barrier had not obviously changed treatment with RP but with RA (Figures 4(a)–4(f)).

### 3.5. HQS-Induced **I**_SC_ Responses in Rat Model

**Figure 5:**
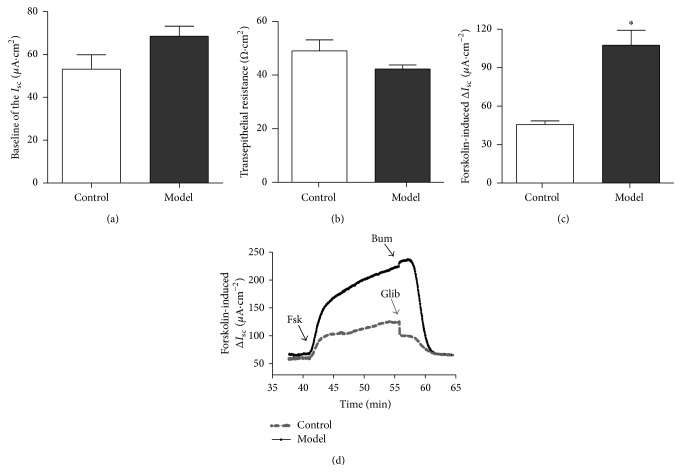
Effects of forskolin-induced *I*_SC_ responses in model rat. The baseline indicates the anion secretion of distal colon in control group and model group (a). The colonic transepithelial resistance has no significant attenuation in the model group (b). Summary of the effects of forskolin- (10 *μ*mol/L, apical) induced Δ*I*_SC_ in model rats distal colon comparison of normal rats (c). Representative *I*_SC_ recording with arrows indicating the time for the apical application of glibenclamide (1 mm/L) or basolateral application of bumetanide (100 *μ*mol/L), respectively, and pretreatment with forskolin (10 *μ*mol/L, apical) (d). Unpaired *t*-test was used. Values are represented as mean ± SEM; ^*∗*^ *P* < 0.05.

In order to further investigate whether HQS has the choleretic action. The model rats had been administered the HQS with 8 g/kg body weight for 6 weeks. As shown in Figures 5(a) and 5(d), the baseline of *I*_SC_ in the model rat is 59.27 ± 6.22 *μ*A/cm^2^ higher than that in the control group 47.46 ± 6.91 *μ*A/cm^2^ (*n* = 8, *P* > 0.05, 19.9%), while in Figure 5(b), the transmembrane resistance in the model is 40.14 ± 2.32 Ω·cm^2^ lower than that in the control group 49.79 ± 2.36 Ω·cm^2^ about 19.90% (*n* = 8, *P* > 0.05). As indicated in Figures 5(c) and 5(d), basolateral addition of forskolin (100 *μ*M) induced a rapid *I*_SC_ rise from 59.27 ± 6.22 *μ*A/cm^2^ to 221.50 ± 24.29 *μ*A/cm^2^ (*n* = 8, *P* < 0.01) in model group, which was higher than in the control group from 45.67 ± 2.86 *μ*A/cm^2^ to 107.50 ± 11.72 *μ*A/cm^2^ (*n* = 8, *P* < 0.01) in model group, respectively. The results indicate that the Δ*I*_SC_ obviously increased after forskolin application was cAMP-dependent on the CFTR expression of the apical side, while basolaterally applied bumetanide, Na^+^-K^+^-2Cl^−^ cotransporter inhibitor (100 *μ*M), or apically applied glibenclamide (1 mM), CFTR channel inhibitor, decreased the forskolin-induced *I*_SC_ response to 53.65 ± 0.09 *μ*A/cm^2^ (Figure 5(d)), which was close to the baseline.

## 4. Discussion

Both human and animal studies have shown that any impairment in the expression and/or function of different hepatobiliary transporters may lead to cholestatic disorders [9]. The impaired secretory function of the biliary epithelium is considered responsible for reduced biliary fluidity and alkalinity for subsequent bile duct damage by cytotoxic compounds or infectious agents [18]. Some experiment results indicate that treatment with ursodeoxycholic acid [19], aimed at improving biliary secretion in terms of bile viscosity and bile acid composition, is currently the most useful therapeutic approach in cystic fibrosis-associated liver disease. Similarly, it was previously shown that tight junctional integrity and transepithelial resistance are relatively resistant to ischemia in bile ducts [20]. The principal effect of HQS is to regulate the functional activities of qi, eliminate stagnation of qi, and enable qi to flow smoothly [15], while Cl^−^ channels have been shown to be important in the regulation of the hepatocyte volume in the presence of altered osmotic conditions; however, the role of this channel in bile flow has not been demonstrated [21].

Electrical parameters, which are monitored in the Ussing chambers, are widely accepted for monitoring the viability and integrity of tissue in the Ussing Chambers. *I*_SC_ reflects the ionic fluxes across the epithelium. In the present study, basal electrical parameters varied over a wide range. This variability has also been observed in previous studies on human tissue samples from jejunum and colon. Formation of bile requires the coordinated function of two epithelial cell types: hepatocytes that are responsible for secretion of the major osmolytes and biliary constituents and cholangiocytes that regulate the fluidity and alkalinity of bile through secretion of osmolytes such as Cl^−^ and HCO_3_^−^ [22]. Studies in isolated cholangiocyte preparations have elucidated the basic transport mechanisms involved in constitutive and stimulated secretory activities in the biliary epithelium. Primary damage to the biliary epithelium is the cause of several chronic cholestatic disorders [23]. From a pathophysiological point of view, common to all cholangiopathies is the coexistence of cholangiocyte death and proliferation and various degrees of portal inflammation and fibrosis. Cholestasis dominates the clinical picture and may initiate or worsen the process. Alterations in biliary electrolyte transport can contribute to the pathogenesis of cholestasis in primary bile duct diseases. Cystic fibrosis-related liver disease represents an example of biliary cirrhosis secondary to a derangement of cholangiocyte ion transport [23].

Epithelial Cl^−^ channels play an important role in regulating and maintaining the normal physiological functions of the GI tract [24]. In this epithelium, Cl^−^ secretion is mediated by two steps, that is, the accumulation of cytosolic Cl^−^ by Na^+^-K^+^-2Cl^−^ cotransporters in the basolateral membrane and then the exit of Cl^−^ through Cl^−^ channels in the apical membrane [25, 26], namely, cystic fibrosis transmembrane regulator (CFTR). Since Cl^−^ secretion requires both the basolateral accumulation of Cl^−^ by Na^+^-K^+^-2Cl^−^ cotransporter and an apical exit through Cl^−^ channels, it is not clear at this point which site is the primary target of HQS. Our results have demonstrated that HQS ethanol extract exerted a stimulatory effect on colon mucosa Cl^−^ secretion by predominantly activating apical cAMP-dependent Cl^−^ channels, namely, CFTR and possibly the Na^+^-K^+^-2Cl^−^ cotransporter [23]. The ability of HQS to stimulate Cl^−^ secretion in the GI tract may contribute to its beneficial effects [27], such as smoothing bowl movement [28] and enhancing fluid clearance during host defense response [29]. It remains uncertain whether the stimulatory effect of HQS on Cl^−^ secretion is due to the collective effect of all the constituent herbal components or some active ingredients contained in the ethanol extract. The general objective of the experiments was to investigate the relation between electrical activity of the intestinal epithelium and capacity to transfer fluid and various solutes. This involved the measurement of the transfer of fluid and solutes by means of short-circuit current. Nevertheless, the current study has established a model for the quantitative measurement of HQS effect on the GI tract to further investigate its possible active ingredients. This effect can potentially improve GI disorders, such as constipation, a condition commonly associated with aged people [30]. The present study demonstrated the stimulation of HQS on the colonic epithelial Cl^−^ secretion. The supporting evidence for the stimulatory effect of HQS on Cl^−^ secretion includes the following: (1) HQS-induced *I*_SC_ increase was insensitive to the Na^+^ channel blocker amiloride but sensitive to Cl^−^ channel blocker glibenclamide and removal of extracellular Cl^−^; (2) the response was inhibited by the inhibitor of Na^+^-K^+^-2Cl^−^ cotransporters, bumetanide. These confirmed the stimulation of Cl^−^ secretion by HQS. Furthermore, Cl^−^ secretion requires both the basolateral accumulation of Cl^−^ by Na^+^-K^+^-2Cl^−^ cotransporter and apical exit through Cl^−^ channels [31]; (3) CFTR, but not *α*-ENaC, in liver, pancreas, and colon, was much higher in the HQS group than those in other groups. As shown in Figures 1, 2, and 4, RA could stimulate the Cl^−^ secretion with the enhancement of *I*_SC_. Our hypothesis is supported by the observation of increased mRNA and protein of CFTR in colon and liver of the treatment group compared with controls. Using multiple methods, these data demonstrate that HQS is inherently altered in alimentary tract CFTR both in* in vitro* colon mucosa and* in vivo* models. These results suggest dominant pathway for regulation of biliary secretion to improve hepatic function [32].

In summary, the results of this study support that HQS and RA can upregulate the expression of CFTR in alimentary tract and evoke colonic ion secretion via CFTR activation, but not *α*-EnaC. Future studies are needed to better delineate this question.

## Figures and Tables

**Table 1:** Sequences of primers.

Primer	Primer sequence	Product length (bp)
CFTR	F: 5′-cgc agg ttc tca gtg gac gat gcc-3′	269 bp
	R: 5′-cct caa cca gaa aaa cca gca cgc-3′	
*α*-ENaC	F: 5′-cag ggt gat ggt gca tgg-3′	239 bp
	R: 5′-cca cgc cag gtc aag-3′	
GAPDH	F: 5′-tgg agt cta ctg gcg tct-3′	401 bp
	R: 5′-agt gag ctt ccc gtt cag-3′	

**Table 2:** Effect of HQS on CFTR and *α*-ENaC protein level in different tissue (mean ± SEM, mm^2^).

Tissue	CFTR			*α*-ENaC		
	Liver	Pancreas	Distal colon	Liver	Pancreas	Distal colon
Control	0.18 ± 0.02	0.14 ± 0.02	0.66 ± 0.11	0.14 ± 0.02	0.05 ± 0.02	0.21 ± 0.04
	(*n* = 6)	(*n* = 6)	(*n* = 6)	(*n* = 6)	(*n* = 6)	(*n* = 6)
RP	0.31 ± 0.03	0.15 ± 0.02	0.68 ± 0.08	0.14 ± 0.00	0.06 ± 0.03	0.25 ± 0.02
	(*n* = 6)	(*n* = 6)	(*n* = 6)	(*n* = 6)	(*n* = 6)	(*n* = 6)
RA	0.34 ± 0.05	0.16 ± 0.03	1.08 ± 0.35^*^	0.15 ± 0.02	0.07 ± 0.03	0.24 ± 0.03
	(*n* = 6)	(*n* = 6)	(*n* = 6)	(*n* = 6)	(*n* = 6)	(*n* = 6)
HQS	0.64 ± 0.22^**^	0.22 ± 0.08^*^	1.05 ± 0.26^*^	0.16 ± 0.04	0.07 ± 0.04	0.24 ± 0.06
	(*n* = 6)	(*n* = 6)	(*n* = 6)	(*n* = 6)	(*n* = 6)	(*n* = 6)

^*∗*^ *P* < 0.05, ^**^ *P* < 0.01 versus control group.

## Abbreviations

HQS: Huqi San
RA: Ramulus Visci alkali
RP: Polysaccharides of Ramulus Visci
CFTR: Cystic fibrosis transmembrane conductance regulator
*α*-ENaC: Epithelial Na^+^ channel
*I*_SC_: Short-circuit current
HBSS: Hank's balanced salt solution
Gli: Glibenclamide
Bum: Bumetanide
Ami: Amiloride.
